# Community Nursing and Cancer Survivorship: A Matter of Appropriateness

**DOI:** 10.1002/nop2.70361

**Published:** 2025-11-10

**Authors:** Martina Torreggiani, Monica Guberti, Deborah Maselli, Stefania Costi

**Affiliations:** ^1^ Research, EBP and IRCCS Unit, Health Professions Department Azienda USL‐IRCCS di Reggio Emilia Reggio Emilia Italy; ^2^ Nursing and Health Professions Directorate IRCCS Istituto Ortopedico Rizzoli Bologna Italy

**Keywords:** cancer survivors, community health nursing, oncology

## Abstract

**Aim:**

We aim to analyse the relationship between a nursing community‐based model of survivorship care, hypothesising its potential application in the Italian context.

**Design:**

Position paper.

**Methods:**

Discussion and agreement on evidence‐based and state‐of‐the‐art knowledge on survivorship care.

**Results:**

The integration of survivorship care in cancer prevention, treatment and rehabilitation pathways is essential to ensure tailored care for patients and their families. Programmes promoting coordinating care between hospitals and communities are promising models of care that may provide more graded assistance. Nurses are promising in this vision, favouring continuity, quality and appropriateness of survivorship care. Survivorship models in Italy are based on multidisciplinary rehabilitating programmes that are geared towards improving quality of life, reducing long‐term complications and preventing cancer relapses. A community nurse‐based model may guarantee equity in access to care by preventing disparities associated with fragilities. Also, it ensures care continuity by delivering services in community facilities. For citizens who are cancer survivors, the community nurse should also be the professional responsible for coordinating the survivorship care plan, promoting appropriateness and tailored care through advanced competence. Integrated into the oncological network, community nurses are capillary, context‐adaptive health ‘facilitators’ within society. Survivorship care models led by community nurses are promising in the Italian context. Organisational studies should be developed to test efficient models for delivering cancer survivorship care.

**Patient or Public Contribution:**

No patient or public contribution.

Cancer is the second leading cause of worldwide disease, with 9.6 million deaths in 2018 (World Health Organization, n.d.‐a). However, the survival rates of several types of cancers are improving thanks to accessible early detection, treatments and survivorship care (World Health Organization, n.d.‐a). The National Cancer Institute's Guidelines on Cancer Survivorship defines cancer survivorship as the life experience of a person with cancer after treatment until the end of life. These guidelines also emphasise how discussing survival problems with carers can give hope to newly diagnosed patients and support them in being proactive towards their treatment path (Sanft et al. 2019). The integration of survivorship care in cancer prevention, treatment and rehabilitation pathways is essential to ensure tailored care for the patient and his family (Ministero della Salute, n.d.). Despite little attention paid to cancer survivorship research in the past years (Ministero della Salute, n.d.), the evidence on this topic has extensively grown, along with the awareness of its relevance (Ministero della Salute, n.d.; Buccafusca et al. 2019). More information on chronic toxicities associated with treatments or health risks correlated to cancer diagnosis would be useful in building effective systems of care (Ministero della Salute, n.d.). In addition, cancer also has an impact on people's social and economic conditions. Regular continuation of employment and career, maintenance of income and the ability to plan must be guaranteed and protected (Ministero della Salute, n.d.). A Firkins et al. (2020) meta‐analysis showed that quality of life was significantly impacted up to 26 years after cancer diagnosis. The current literature highlights a substantial heterogeneity in the unmet needs of cancer survivors (CSs), with informational, financial and physical needs being the most prevalent (Hart et al. 2022; Fitch et al. 2021). Mood disturbances, such as distress, depression and anxiety, are factors frequently associated with the presence of unmet needs (Hart et al. 2022). Evidence‐based education programmes on cancer survivorship are warranted for providers (Chan et al. 2022) who must rely on valid tools for an accurate assessment of unmet needs and an appropriate resource allocation (Contri et al. 2023). Patient‐centred programmes promoting coordinating and dynamic care between hospital and community are promising models of care, which may ensure more graded assistance (Torreggiani et al. 2024), and nurses are promising in this vision, favouring continuity, quality and appropriateness of survivorship care.

This critical reflection aims to analyse the relationship between a nursing community‐based model of survivorship care, hypothesising its potential application in the Italian context.

In Italy, from 2018 to 2023, the 5‐year cancer survival for all cancers increased compared to previous data (Associazione Italiana Registri Tumori, n.d.). The strategies to be adopted in providing care for CSs recognise the value of survivorship care models based on multidisciplinary follow‐up programmes that include specialists, nurses, general practitioner rehabilitation professionals, psychologists and social workers (Ministero della Salute, n.d.). Moreover, social policies that support the return to work, involving voluntary associations and e‐health, are suggested (Ministero della Salute, n.d.). These models are based on effective communication between patients and professionals (Ministero della Salute, n.d.). The care of CSs should be oriented towards rehabilitating the individual, which includes returning to relevant roles and occupations and the right to be forgotten (Dal Maso et al. 2022; Ustrell 2023). Furthermore, healthy lifestyles should be promoted to improve quality of life through the reduction of long‐term complications of cancer and also the prevention of cancer relapses.

Among the existing models of survivorship care, those that are based on community nurses guarantee (1) equity in access to care by preventing disparities associated with fragilities and intersectionality and (2) care continuity by the delivery of services in community facilities (Ministero della Salute, n.d.; Dal Maso et al. 2022). For citizens who are CSs, the community nurse should also be the professional responsible for coordinating the survivorship care plan, which is tailored to the individual's needs. These models also ensure appropriateness and quality of care through advanced competence. For instance, remote or home monitoring reduces the number of hospital admissions and anticipates detecting toxicity, with definite advantages, especially in the elderly population (Ministero della Salute, n.d.; Dal Maso et al. 2022; Rogers et al. 2023). Back in 1998, the World Health Organization first outlined community nurses' role for the first time (World Health Organization, n.d.‐b). In Italy, with the DL 77/2020, the professional figure is legally defined, which ensures nursing care at different levels of complexity in collaboration with the entire multidisciplinary team in the community of reference. The community nurse represents a competent facilitator in most health fields, both for patients and caregivers. This healthcare professional may have a global vision of the population's needs with particular attention to the most fragile subjects, operating in close relationships with the general practitioners, social and welfare services, families, carers and specialists (LEGGE 17 Luglio 2020, n. 77, n.d.). In 2022, a Ministerial Decree (77/2022) establishes models and standards for developing community care in the National Health Service, including the community nurse. This professional should be present for every 3000 inhabitants (MINISTERO DELLA SALUTEDECRETO 23 Maggio 2022, n. 77, n.d.).

Pivotal for the healthcare objectives of the community nurse is knowing the context in which the patient lives since this professional plays a fundamental role in health promotion and intervening in the environment through education and prevention (Conferenza delle Regioni, Documento Recante: ‘Linee di Indirizzo Infermiere di Famiglia/Comunità’ ex L. 17 Luglio 2020 n. 77). The community nurse is, therefore, the one who, in synergy with the other professionals, helps individuals to adapt to the disease and chronic disability, both at the patient's home and in health facilities that insist in the community settings (Conferenza delle Regioni, Documento Recante: ‘Linee di Indirizzo Infermiere di Famiglia/Comunità’ ex L. 17 Luglio 2020 n. 77; World Health Organization 2021). This professional contributes to setting the standards for the best assistance tailored for each case. The patient should be guaranteed an appropriate clinical reference to services in the community at every stage of the follow‐up. Nonetheless, the follow‐up should be differentiated, with CSs with low‐risk profiles falling within the scope of the general practitioner's activities and medium‐high‐risk CSs with followed‐up oncology specialists performing activities in outreach facilities (Rodriguez et al. 2023).

Context is a determinant of the success of a new service introduction. Referring to the Emilia‐Romagna region, structural, organisational and technological standards are defined for oncological and haematological activities, including community healthcare facilities, different healthcare professionals involved and the necessary skills (Salute Regione Emilia Romagna, n.d.). The oncological network is developed in different care settings, from the acute hospital to the community. A pivotal objective of this model, called ‘proximity oncology’, is to guarantee vulnerable patients' fair access to health services (Prevenzione DGR‐Cancro‐2029_2023‐.pdf).

The organisational model of Emilia‐Romagna is the Comprehensive Cancer Care Network (CCCN). The CCCN is divided into three operational levels (Salute Regione Emilia Romagna, n.d.):

- Level I centres: Territorial structures, such as hospitals/community homes where oncological health services are implemented (follow‐up, advice).
- Level II centres: District hospitals can provide diagnostic, therapeutic and care services.
- Level III centres: Multispecialty hospitals in which all the specialist skills of oncological and haemato‐oncological interest are present, high‐complexity technological equipment is used and continuing training and research programmes are offered, such as the Research and Scientific Care Hospitals (IRCCS).

In this context, we hypothesise that community nurses are well‐suited to serve as survivorship care coordinators. Their ability to integrate services from both the community and hospital settings would ensure optimal care that aligns with the needs of CSs. However, to verify this assumption, the scientific community should implement organisational studies to develop efficient models for delivering cancer survivorship care.

In Conclusion: Community nurses, fully integrated into the oncological network, are capillary, context‐adaptive health ‘facilitators’ within society. Survivorship care models led by community nurses, who work in close relationships with other health and social providers, are promising models for providing survivorship care in the Italian context.

## Ethics Statement

The authors have nothing to report.

## Consent

The authors have nothing to report.

## Conflicts of Interest

The authors declare no conflicts of interest.

## Data Availability

The authors have nothing to report.
